# Dialysis therapy for Syrian refugees in Lebanon: a call for action

**DOI:** 10.2471/BLT.18.214866

**Published:** 2018-06-01

**Authors:** Nabil Karah, Mohamed Nour Kurhani, Eyad Fadloun, Bahija Mayasi, Federico Orioli, Kristil Haraldstad, Erik Fosse

**Affiliations:** a Norwegian Aid Committee, Arbeidersamfunnets Plass 1, 0181 Oslo, Norway.; b Union of Relief and Development Associations, Beirut, Lebanon.; c Health Care Society, Beirut, Lebanon.

The Syrian refugee crisis, now in its eighth year, is the largest displacement crisis of our time. With about 1 million registered refugees, whether nationals or permanent residents, from the Syrian Arab Republic, Lebanon currently has the highest number of refugees per capita in the world.^1^ Syrian refugees with chronic diseases face challenges in Lebanon, because of the high costs of health-care services, restrictions on work and mobility and insufficient funds for humanitarian aid. Interruptions in the routine health care of patients with chronic diseases put them at high risk of serious complications or death.^2^ While the United Nations Refugee Agency (UNHCR) provides a wide range of health-care services to registered Syrian refugees in Lebanon, meeting the cost of recurring hospital treatments for chronic diseases, such as cancer and renal failure, is beyond the capacity of UNHCR.^3^

In October 2015, the Norwegian Aid Committee launched a programme to support Syrian refugees in Lebanon, with a special focus on patients with chronic renal failure, thalassemia, sickle cell anaemia, haemophilia or other non-malignant chronic blood diseases. Since then, the programme has been extended yearly, with the current phase ending in February 2019. The programme is implemented in partnership with two Lebanese nongovernmental organizations, the Union of Relief and Development Associations and the Health Care Society, in coordination with UNHCR and the Syrian American Medical Society. All medical consultations, treatment sessions and laboratory tests are carried out through the Lebanese health-care system.

As of March 2018, based on a country-wide assessment, the Norwegian Aid Committee, Union of Relief and Development Associations, and Health Care Society, had located a total of 218 patients from the Syrian Arab Republic, 206 Syrians and 12 Palestinians, who need regular treatment for chronic renal failure. Of these, 128 (59%) are males and 90 (41%) females; 54 (25%) are settled in north Lebanon, 57 (26%) in Beirut and Mount Lebanon, 76 (35%) in the Bekaa and 31 (14%) in the South and Nabatiye. Therapy is provided in 27 kidney dialysis facilities throughout Lebanon. The Norwegian Aid Committee programme supports 114 (52%) of these patients. The remaining patients either receive assistance from other nongovernmental organizations, including the Syrian American Medical Society, Kuwait Red Crescent Society and Caritas Lebanon or cover their own treatment costs. Since some funds might run out this year,^3^ many of these patients risk losing access to their dialysis sessions, unless additional funding is secured. Initiatives, such as the fundraising gala recently organized by the Syrian American Medical Society, are urgently needed.^4^

For the 114 patients assisted through this programme, the average cost of a dialysis session has been around 110 United States dollars (US$), with an average monthly cost of US$ 1320 per patient. These patients cannot bear such financial burden. Moreover, the influx of Syrian refugees has already overstretched the capacity of the highly privatized health-care system in Lebanon.^5^^,^^6^ Disruption of dialysis sessions might lead to a substantial increase in deaths;^7^ however, current interventions, including our programme, are not able to cover all the needs for the management of chronic renal failure among the refugees in Lebanon. A recent survey on the medical and psychosocial challenges of 57 Syrian refugees who required dialysis in Jordan, showed that 14 patients (25% of those surveyed) had at least one period of dialysis interruption of a week or more, with financial reasons being the most common cause.^8^ The survey highlighted the need for bold actions, such as asking patients to pay a fraction of their dialysis cost.

The Norwegian Aid Committee calls on stakeholders and UNHCR to develop an action plan for the long-term management of chronic renal failure among Syrian and Palestinian refugees from the Syrian Arab Republic in Lebanon. The plan should be based on a systematic evaluation of the medical needs and resources, and include input from a monitoring panel of nephrologists and beneficiaries. Relevant private sector actors, such as hospital managers and dialysis companies, should also be engaged in identifying the most cost-effective conditions for the plan’s success. Generous funding from donors is needed to enable humanitarian organizations to continue offering dialysis services. A predictable fund flow would enable the Norwegian Aid Committee and other partners to manage needs and better plan their future actions.
